# Detection and Identification of Estrogen Based on Surface-Enhanced Resonance Raman Scattering (SERRS)

**DOI:** 10.3390/molecules23061330

**Published:** 2018-06-01

**Authors:** Yang Liu, Yue Chen, Yuanyuan Zhang, Qiangwei Kou, Yongjun Zhang, Yaxin Wang, Lei Chen, Yantao Sun, Honglin Zhang, Young Mee Jung

**Affiliations:** 1 College of Physics, Jilin Normal University, Siping 136000, China; liuyang@jlnu.edu.cn (Y.L.); 17649973053@163.com (Y.C.); 13944139606@163.com (Y.Z.); 13944949603@163.com (Q.K.); zhangyongjunwyx@126.com (Y.Z.); wangyaxin1010@126.com (Y.W.); syt@jlnu.edu.cn (Y.S.); 2 Key Laboratory of Functional Materials Physics and Chemistry of the Ministry of Education, Jilin Normal University, Changchun 130103, China; 3 School of Electronic and Information Engineering, South China University of Technology, Guangzhou 510640, China; eezhl@scut.edu.cn; 4 Department of Chemistry, Institute for Molecular Science and Fusion Technology, Kangwon National University, Chunchon 24341, Korea

**Keywords:** phenolic hormones, SERRS, detection

## Abstract

Many studies have shown that it is important to consider the harmful effects of phenolic hormones on the human body. Traditional UV detection has many limitations, so there is a need to develop new detection methods. We demonstrated a simple and rapid surface-enhanced resonance Raman scattering (SERRS) based detection method of trace amounts of phenolic estrogen. As a result of the coupling reaction, there is the formation of strong SERRS activity of azo compound. Therefore, the detection limits are as low as 0.2 × 10^−4^ for estrone (E1), estriol (E3), and bisphenol A (BPA). This method is universal because each SERRS fingerprint of the azo dyes a specific hormone. The use of this method is applicable for the testing of phenolic hormones through coupling reactions, and the investigation of other phenolic molecules. Therefore, this new method can be used for efficient detection.

## 1. Introduction 

Estrogen, which is also known as a “female hormone”, is a natural hormone and steroid that has very important physiological roles [1,2]. However, researchers have found that the use of synthetic estrogen or estrogen-like compounds is related to the development of certain diseases, such as breast cancer, which has attracted considerable interest [3,4]. This type of hormone can increase the incidence of breast cancer, heart disease, stroke and thrombosis. Thus, estrogen can be considered a carcinogen [5,6,7]. Therefore, it is very important to maintain the hormone concentration within safety standards and to develop rapid and highly sensitive detection methods of estrogens.

At present, there are two types of estrogen detection methods: direct and indirect detection. The direct detection method of estrogen includes liquid chromatography–mass spectrometry (LC–MS) [8,9] and high performance liquid chromatography–tandem quadrupole mass spectrometry (HPLC–MS/MS) [10,11]. The objectives of Bogg’s study was to develop a liquid chromatography–tandem mass spectrometry (LC–MS/MS) method for multi-class steroid hormone detection using biologically relevant concentrations, before testing limits of detection (LOD) in a high-background matrix by spiking charcoal-stripped fetal bovine serum (FBS) extract [12]. Čelić et al. used an online ultra-high-performance-liquid chromatography–triple quadrupole tandem mass spectrometry (UHPLC–MS/MS) method for detection and quantification of natural and synthetic estrogens and their conjugates in aqueous matrices [13]. However, these methods have some limitations as the sample pretreatment process is relatively complex, the testing time is relatively long and the results from these methods cannot be widely applied. On the other hand, the indirect detection method, such as the high pressure liquid chromatography–radioimmunoassay (HPLC–RIA) [14], enzyme-linked immunosorbentassay [15] and plasma biosensor [16], has high sensitivity. Nevertheless, these methods require complex antibody preparation and blurring in specific and non-specific recognition boundaries. Therefore, the development of a simple, rapid, highly sensitive and selective detection method of estrogen has become the top priority of researchers.

Surface-enhanced Raman spectroscopy (SERS) is a highly sensitive analytical technique that enables the detection of single molecules [17,18,19,20,21]. As an ultrasensitive method, SERS has been widely used in the determination of chemicals and biological molecules [22,23,24]. The SERS study for estrogen is focused on improving the adhesion of these estrogens to Ag (or Au) surfaces, thereby enhancing the SERS. For the detection of estrogen based on SERS, one of the most important issues is the direct connection of phenolic estrogens and Ag (or Au) with specific difficulties [25,26]. Therefore, the interaction between Ag (or Au) and estrogen has been intensively studied. The selectivity and sensitivity of this method can be further improved by combining the surface enhancement with the molecular resonance Raman (RR) effect (i.e., the excitation line in resonance with both the SPR and electronic transition of the close molecules). This approach has been called the surface-enhanced resonance Raman scattering (SERRS) [27,28].

There is a coupling reaction between the phenol and diazonium ions, which produces products known as azo dyes. Thus, Pauly’s reagent is often used for tyrosine detection based on the optical absorption of the products, azo compounds. These azo compounds have been proven to have strong Raman activity and a propensity toward binding to Ag NPs [29]. Coupled with surface-enhanced resonant Raman scattering (SERRS), the SERRS “fingerprint” spectra of different azo dyes correspond to the detection and identification of different phenolic estrogens.

In this study, we investigated a sensitive SERS-based detection method of phenolic estrogens. To greatly enhance the adhesion of analytes to the surface of metal nanoparticles as well as the SERS signal, we used the coupling reaction between phenolic estrogens and Pauly’s reagents. This proposed method has the following advantages. First, it is very simple as it only involves the mixing of estrogen, Pauly’s reagent and silver colloid without the need for a separation or purification procedure. Second, it takes about 2 min to complete the coupling reaction and SERS measurement, which allows for rapid analysis. Third, the ultra-sensitive detection was realized using SERS. These results indicate that this detection method shows significant potential in being highly sensitive for most molecules with phenolic groups due to the phenolic coupling reaction.

## 2. Experimental Section

### 2.1. Materials

**Figure 1:**
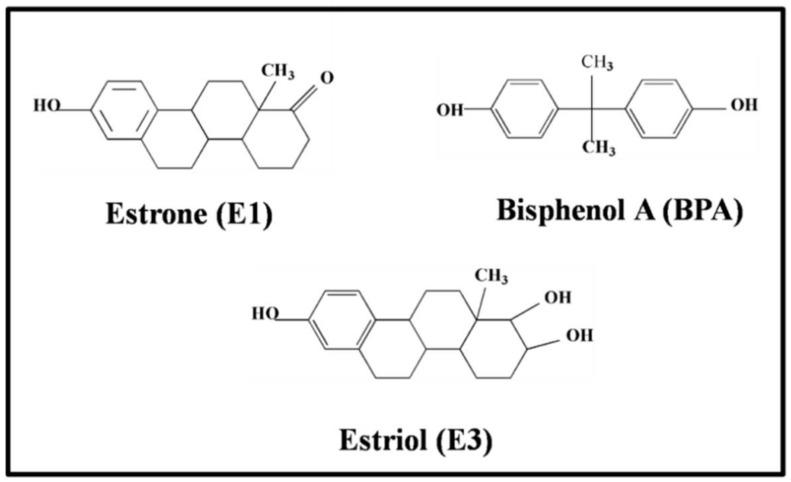
Molecular structures of two natural estrogens (estrone (E1), and estriol (E3)) and a synthetic estrogen (bisphenol A (BPA)).

Estrone (E9750, E1), estriol (E1253, E3) and bisphenol A (239658, BPA) were the three estrogens used in this present study, with their structures shown in Figure 1. Silver nitrate was purchased from Sigma-Aldrich Co., Ltd., (Shanghai, China), at the highest purity available and used as received without further purification. The estrogens were dissolved in ethanol or dioxane. Infant formula was purchased from Meiji Co., Ltd., (Shanghai, China). Ultrapure water (18.0 MΩ cm^−1^) was used throughout the present study.

#### 2.1.1. Preparation of Silver Nanoparticles

Colloidal silver nanoparticles were prepared by the aqueous reduction of silver nitrate (10^−3^ M, 200 mL) with trisodium citrate (1%, 4 mL) using the method described by Lee and Meisel [30]. The colloidal silver nanoparticles showed a maximum absorption at 420 nm.

#### 2.1.2. Pauly’s Reagent and Coupling Reaction

We prepared three reagents. Reagent A was prepared by dissolving 4.5 g of *p*-aminobenzenesulfonic acid in 500 mL of deionized water, before adding 5 mL of 12 M HCl (the solution was stored at 4 °C). Reagent B was a 5% sodium nitrite solution (the solution was stored at 4 °C) and reagent C was a 10% sodium carbonate solution. The coupling reagent configuration was reagent A, reagent B, reagent C and estrogen in a volume ratio of 1:1:1:2.

#### 2.1.3. SERS Measurement

After the coupling reaction, 25 μL of each sample and an equal amount of colloidal silver nanoparticles were added drop wise to the aluminum pan (No. 0219-0041, Perkin-Elmer, Waltham, MA, USA before the mixture was irradiated with a laser beam for 30 s before each SERS measurement. The SERS spectra were measured using a Renishaw Raman microsystem 2000 equipped with a charge-coupled device (CCD) detector and a holographic notch filter. A wavelength of 514.5 nm Ar^+^ ion laser (Spectra Physics, Harwell, UK) was used as an excitation source for SERS measurement. The typical exposure time used during this study was 10 s with 15-mW power directed at each sample.

#### 2.1.4. Characterization Methods

A detailed structure of the obtained sample was obtained by a JEOL 2100 transmission electron microscope (TEM) operating at 200 kV. The light scattering experiments (both SLS and DLS) were performed using the Zetasizer Nano instrument (Malvern, UK). Ultraviolet–visible spectroscopy (UV–Vis) absorbance spectra were recorded on a Shimadzu UV 3600 spectrophotometer. The SERS spectra were measured using a Renishaw Raman microsystem 2000 equipped with a charge-coupled device (CCD) detector and a holographic notch filter. A wavelength of 514.5 nm Ar^+^ ion laser (Spectra Physics) was used as an excitation source for SERS measurement.

## 3. Results and Discussion

Two natural steroid estrogens (estrone (E1) and estriol (E3)) and a nonsteroidal estrogen (bisphenol A (BPA)) as a typical hormone were used in this study. The molecular structures of these estrogens are shown in Figure 1. As shown in Figure 1, the molecular structures of two natural estrogens are quite similar. The fundamental difference between these two natural steroid estrogens is the number of hydroxyl groups: E1 has a hydroxyl group and E3 has two hydroxyl groups. In contrast, the nonsteroidal estrogen BPA has a symmetrical structure. As mentioned earlier, the affinity of these phenolic estrogens to the silver nanoparticles is weak, which leads to a great reduction in the SERS sensitivity.

**Figure 2:**
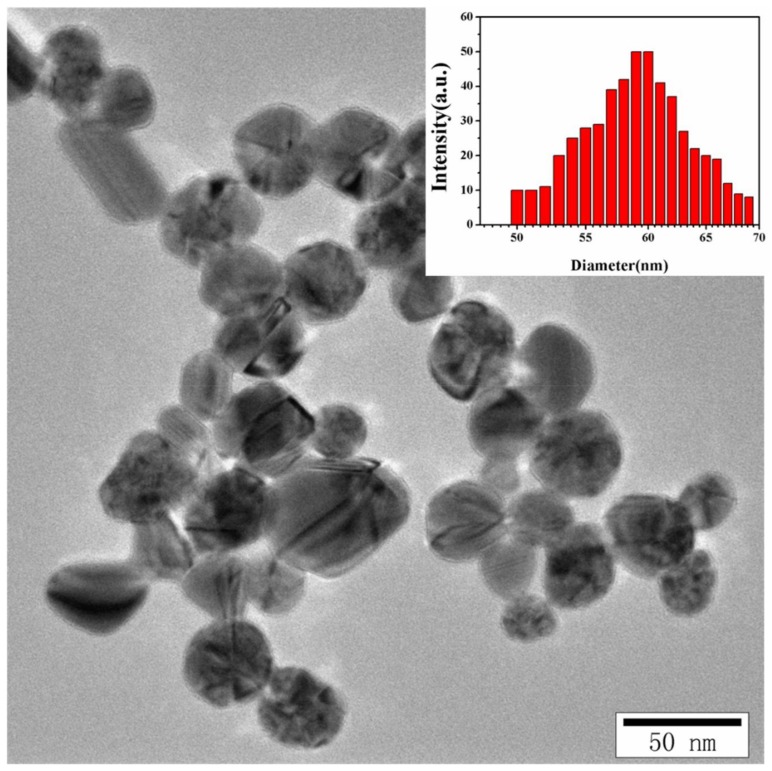
TEM images of the pure Ag NPs with the distribution of hydrodynamic sizes of Ag NPs (inset).

In fact, the pure silver colloids still have good dispersibility and demonstrate a narrow particle size distribution, which is shown in Figure 2. The corresponding average particle size in average is 41 nm. The particle size of the nanocrystals can also be determined by measuring the random changes in the intensity of light scattered from a suspension or solution. The dynamic light scattering (DLS) is used to analyze the particle size using the associated distribution of the as-prepared Ag NPs, which is depicted in the inset of Figure 2. From the size distribution histograms obtained via DLS measurement, the average size of Ag NPs abnormally reaches up to ~70 nm. The abnormal increase in the average diameter as demonstrated by DLS measurements is mainly attributed to the aggregation of Ag NPs in aqueous solution [31]. Although we utilized the dried form of the synthesized samples in TEM, the size of the samples was estimated in an aqueous dispersion in DLS, which provides the hydrodynamic diameter of the samples. Furthermore, DLS gives an overall size distribution, but the size of the aggregation is usually measured instead of the individual nanoparticles. Therefore, the size obtained from the DLS measurement tends to be larger than that of TEM as DLS takes the aggregates and surfactants into account. 

**Figure 3:**
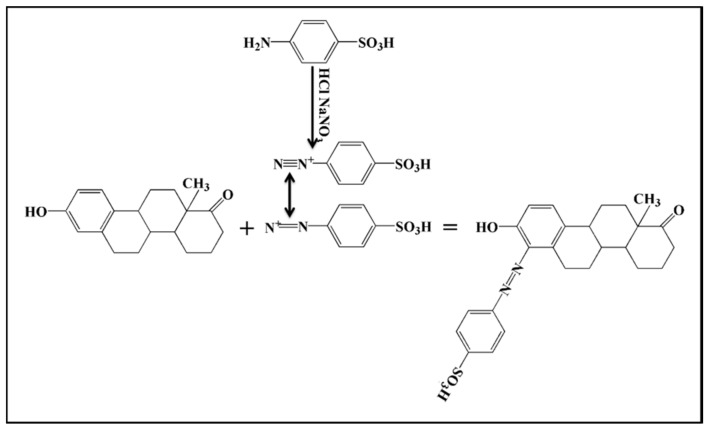
Coupling reaction between estrone (E1) and diazonium ions.

To improve the affinity of estrogen and silver nanoparticles, we used the azo reaction, which is shown in Figure 3. This azo reaction can further enhance SERS. A coupled reaction is a process in which two organic chemical units (hydrocarbon fragments) are coupled with the aid of a metal catalyst to obtain an organic molecule. In coupling reactions, the acidity medium is very important. In Figure 3, Azo-N = N- is linked to a hydroxyl group. The R-N = N-R’ azo compounds have cis and trans isomers, with the trans isomer being more stable than the cis isomer. The two isomers can be converted to each other under light or heating conditions. Azo compounds are a class of colored compounds, some of which can be directly used as a dye or indicator. In organic analysis, the use of coupling reactions to produce colors can identify phenolic or aromatic amine structures.

**Figure 4:**
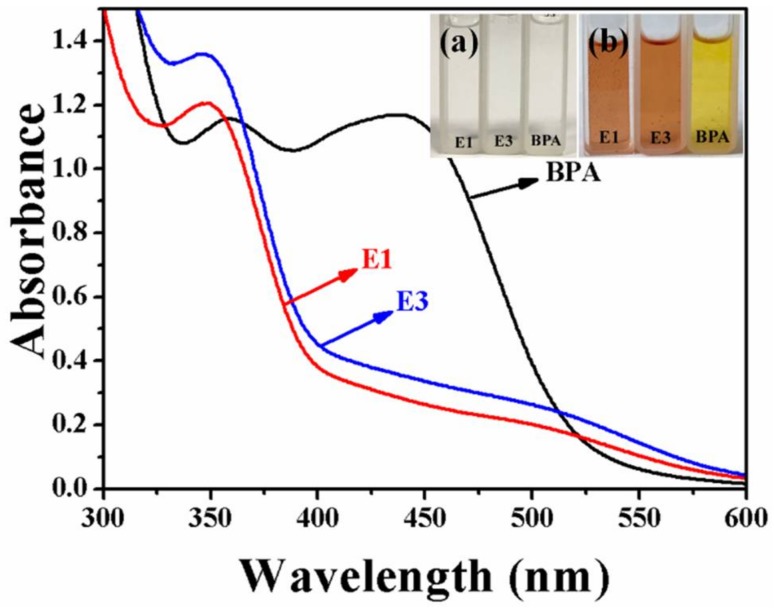
Visual color change before (a) and after (b) coupling reactions between the estrogens E1, E3 and BPA; and the UV–Vis spectra of the corresponding azo dyes.

As a result, when estrogen is added to Pauly’s reagent, we observed a significant change in color (the inset of Figure 4). When observing with the naked eye, E1 and E3 turn orange, while BPA and other substances turn yellow after the reaction with Pauly’s reagent. The color changes were more clearly distinguished by UV–Vis spectroscopy (Figure 4). We noted that two of the azo dyes had a similar absorption at ∼500 nm, which differed from that of the azo product from BPA (∼450 nm) [26]. When E1, E3 and BPA reacted with the azo dye with the subsequent addition of silver, we found that the absorption was still around ∼500 nm, because the absorption peak of Ag was coupled with the absorption peak of estrogens (see Figure S1 in the Supplementary Materials). Therefore, 514.5-nm laser excitation was selected for all estrogens, which was chosen according to the resonance effect of the samples and the plasmon resonance of silver nanoparticles with the laser. The UV–Vis detection of hormones has the disadvantage of a relatively low sensitivity and, thus, we chose the SERRS method for testing. This method that is based on the azo coupling reaction and SERRS technique has the following advantages of being: (1) simple and fast as the azo-Ag mixture for SERRS test is very easy to prepare, which only requires direct mixing and can be completed in 2 min; and (2) practical and reliable in the detection of phenolic endocrine disruptors.

**Figure 5:**
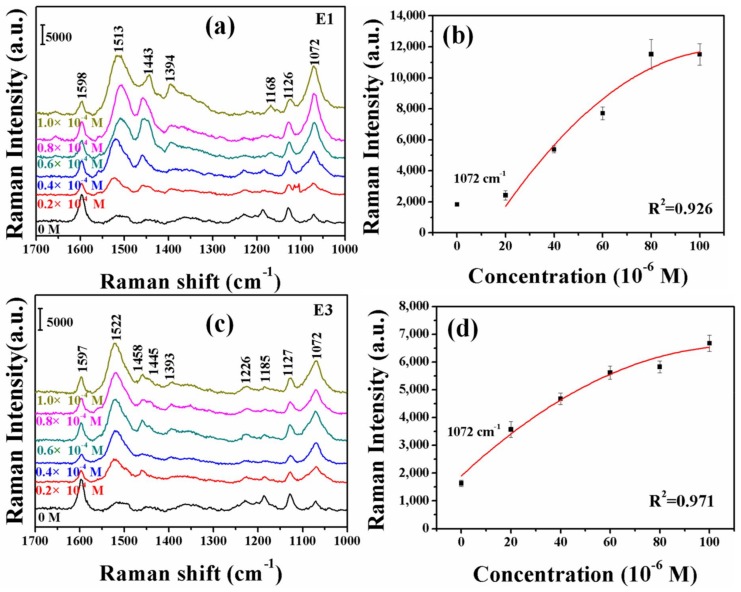
Raman test results after azo reaction of E1 (**a**) and E3 (**c**) at concentrations of 1.0 × 10^−4^ M, 0.8 × 10^−4^ M, 0.6 × 10^−4^ M, 0.4 × 10^−4^ M, 0.2 × 10^−4^ M and 0 M, respectively. Concentration-dependent SERRS intensities of the Raman bands at 1072 cm^−1^ for E1 (**b**) and E3 (**d**).

Compared with the UV–Vis spectra, the Raman spectra have a significant advantage as the high-resolution structure exhibited excellent quantitative and qualitative applications. This characteristic property encouraged us to select SERS as the basic research method. Compared with the traditional UV–Vis detection, the sensitivity is greatly improved. The estrogen fingerprint information can be observed by using the SERS technology (Figure 5). Moreover, the normal Raman and SERS spectra of diazonium ions before reacting with phenolic estrogens are very weak, which almost have no interference with SERS spectra of the estrogen-derived azo dyes (see Figure S2 in the Supplementary Materials). The concentration-dependent SERRS intensity of the bands at 1072 cm^−1^ for E1 and E3, respectively, adsorbed on Ag substrate is shown in Figure 5. At all frequencies, the SERS intensity varies monotonously with a change in concentrations.

**Figure 6:**
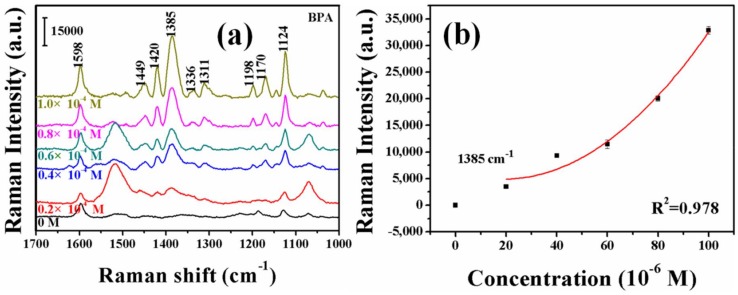
(**a**) Raman spectra after azo reaction of BPA at the concentrations of 1.0 × 10^−4^ M, 0.8 × 10^−4^ M, 0.6 × 10^−4^ M, 0.4 × 10^−4^ M, 0.2 × 10^−4^ M and 0 M, respectively; and (**b**) the plot of the SERS intensity of the band at 1385 cm^−1^ for BPA as a function of the concentration.

From the concentration-dependent SERS spectra of BPA shown in Figure 6a, we found that, when the concentration of BPA was 0.2 × 10^−4^ M, there was one vibrating band at 1420 cm^−1^, which was mainly attributed to the –N=N– stretching vibration of the trans isomer. Compared with the other SERS spectra of the estrogen-derived azo dyes investigated in this study, the SERS spectra of the BPA-derived azo dye is unique, due to the presence of a weaker band at 1449 cm^−1^ and a new band at 1420 cm^−1^, which probably originated from the stereo structure of BPA and its steric effects on –N=N– stretching. Moreover, with an increase in the BPA concentration, the intensity of these two bands gradually increases. The bands at 1598, 1385 and 1124 cm^−1^ may originate from the –C–C– within phenol and/or phenyl rings. Furthermore, a band at 1385 cm^−1^ also comes from the phenol groups in the –CH– and –OH vibration. A band at 1198 cm^−1^ is the result of the effects of –CH, –OH and –C–C in the phenol group in –CN and phenyl-N in –C–N–N [32,33,34,35]. The concentration dependent SERRS intensity at the 1385 cm^−1^ band for BPA adsorbed on Ag substrate is shown in Figure 6b. The SERS intensity of all bands varies monotonically with a change in concentrations.

**Figure 7:**
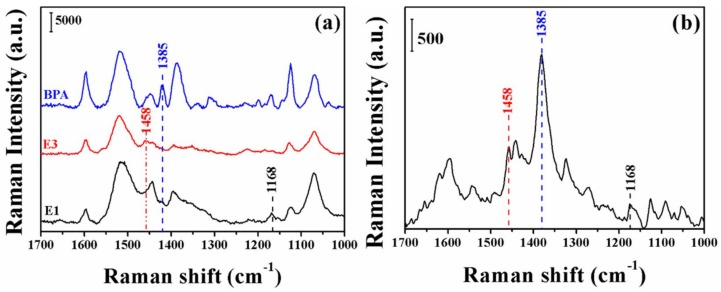
SERRS spectra of the estrogen-derived azo dyes from estrogen mixtures (E1, E3 and BPA) (**b**); and their individual spectra (each estrogen was at a concentration of 1.0 × 10^−4^ M) (**a**).

To investigate the reusability of the proposed method, we studied the spectrum of SERRS azo dyes in estrogen mixtures. Figure 7a shows the SERRS spectra of the three hormones E1, E3 and BPA separately, while Figure 7b shows the SERRS spectra of the three hormone cocktails. Due to their azo structure, the affinity of the azo dyes for their silver nanoparticles allows the parallel adsorption of overlapping SERRS bands. The 1168, 1485 and 1385 cm^−1^ bands in Figure 7b correspond to the characteristic peaks of E1, E3 and BPA in Figure 7a, respectively. As shown in Figure 7b, composite SERRS bands were clearly observed in the azo dyes of the estrogen mixture, confirming the application potential of the present method for the detection of an estrogen mixture. 

## 4. Conclusions

In summary, this SERRS-based detection method significantly improves the sensitivity of the detection of phenolic hormones. The use of coupling reactions to form a “bridge” between Ag and phenolic hormones is an indirect means, which provides the advantages of being simple and fast with universal applications and high selectivity. This can be used for the sensitive and rapid detection of other phenolic hormones. Our approach provides a simple, fast and ultra-sensitive platform for the detection of phenolic estrogen in practical applications. It can be extended to numerous other applications in the food safety and environmental safety fields.

## Supplementary Materials

The following are available online. Figure S1: The UV–Vis spectrum of silver colloids added after the coupling reaction of phenol and diazo ion. Figure S2: Raman spectra of pure E1 dissolved and its SERRS spectra in silver colloid; the concentration of E1 was 100 ppm.
