# Supplementary material for: Detection and Identification of Estrogen Based on Surface-Enhanced Resonance Raman Scattering (SERRS)
Source: Molecules. 2018 Jun 1;23(6):1330. doi: 10.3390/molecules23061330 (PMC6099535; doi:10.3390/molecules23061330)
Supplement: Supplementary file 1 [file molecules-23-01330-s001.pdf]

Detection and Identification of Estrogen Based on Surface-enhanced Resonance Raman Scattering (SERRS)

Yang Liu[1, 2], Chen Yue[1, 2], Yuanyuan Zhang[1, 2], Qiangwei Kou[1, 2], Yongjun Zhang[1, 2], Yaxin Wang[1, 2], Lei Chen[1, 2*], Yantao Sun[1, 2], Honglin Zhang[3], Young MeeJung[4*]

[1] College of Physics, Jilin Normal University, Siping 136000, China.

[2] Key Laboratory of Functional Materials Physics and Chemistry of the Ministry of Education, Jilin Normal University, Changchun 130103, China.

[3] School of Electronic and Information Engineering, South China University of Technology, Guangzhou, 510640, China.

[4] Department of Chemistry, Institute for Molecular Science and Fusion Technology, Kangwon National University, Chunchon 24341, Korea.

Abstract

A large number of studies have shown that the harmful effect of the phenolic hormones on the human body should be considered. Traditional UV detection has many limitations, so it is urgent to develop new detection methods. We demonstrate a simple and rapid surface-enhanced resonance Raman scattering (SERRS) based detection method of trace amounts of phenolic estrogen. As a result of the coupling reaction, the formation of a strong SERRS activity of azo compounds, therefore, the detection limits are as low as 0.2×10^{-4}, 0.4×10^{-4}, 0.6×10^{-4}, 0.8×10^{-4}, and 1.0×10^{-4} M for estrone(E1), estriol (E3), and bisphenol A (BPA). This method is universal because each SERRS fingerprint of the azo dye corresponds to its own corresponding hormone. The use of this method not only is applicable to test phenolic hormones through coupling reactions, but also to investigate other phenolic molecules. Therefore, this method can be used as a new type of efficient detection one.

Keywords: Phenolic hormones; SERRS; Detection.

* Corresponding authors: chenlei@jlnu.edu.cn; ymjung@kangwon.ac.kr

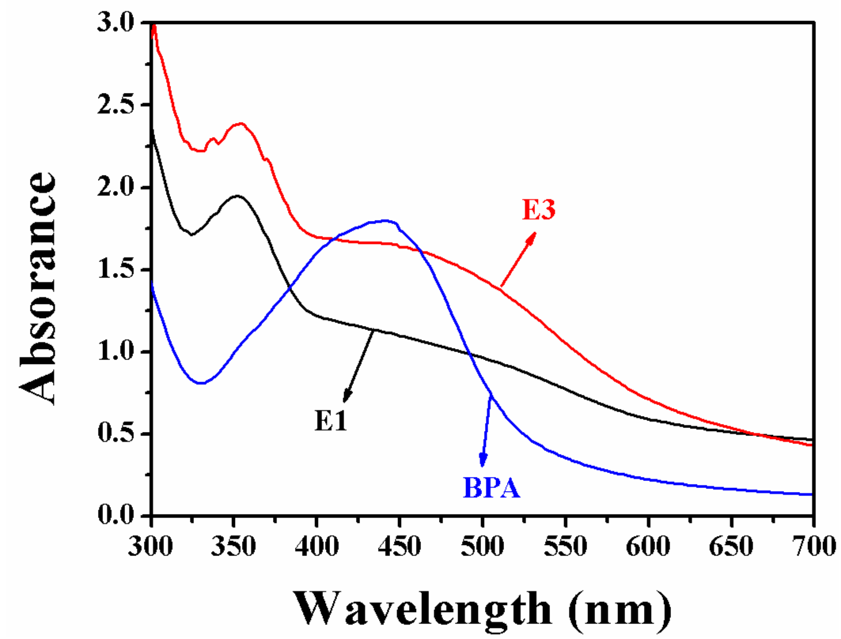

S1. The UV/Vis spectrum of silver colloids added after coupling reaction of phenol and diazo ion.

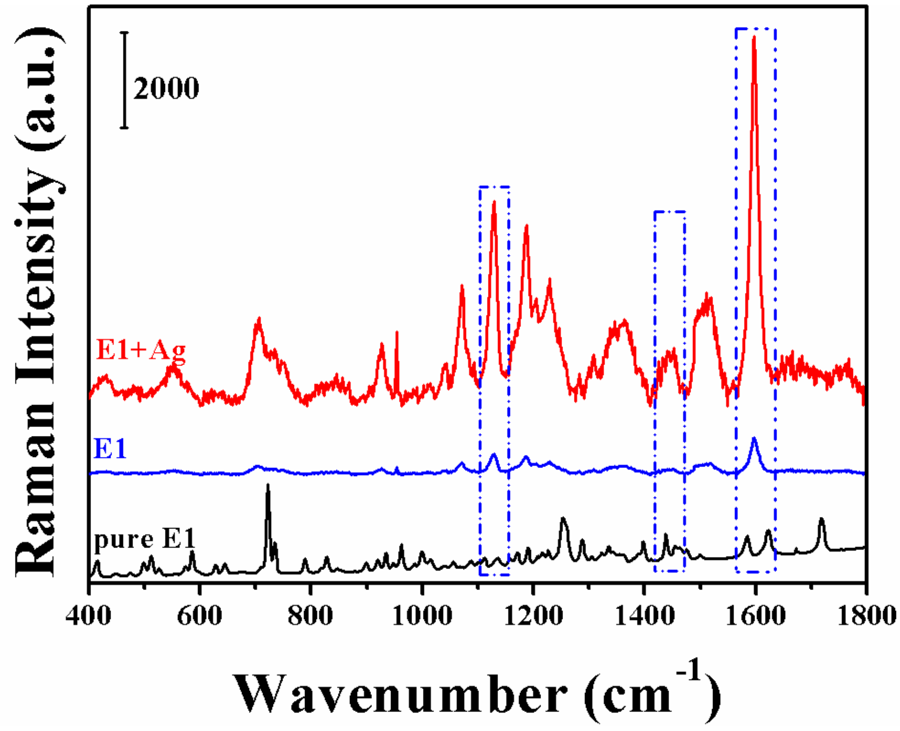

S2. Raman spectra of pure E1, dissolving in ethanol and its SERRS spectra in silver colloid; concentration of E1 was 1.0×10^{-4} M.
